# Case report: Dancing in the dark: A critical single case study engaging a blind father in the rehabilitation journey of his visually impaired child

**DOI:** 10.3389/fpsyg.2022.942321

**Published:** 2022-10-13

**Authors:** Livio Provenzi, Giada Pettenati, Antonella Luparia, Daria Paini, Giorgia Aprile, Federica Morelli, Eleonora Mascherpa, Luisa Vercellino, Serena Grumi, Sabrina Signorini

**Affiliations:** ^1^Department of Brain and Behavioral Sciences, University of Pavia, Pavia, Italy; ^2^Developmental Psychobiology Lab, IRCCS Mondino Foundation, Pavia, Italy; ^3^Developmental Neuro-Ophthalmology Unit, IRCCS Mondino Foundation, Pavia, Italy

**Keywords:** blind parent, visually impaired children, state space grid, parent-child interaction, dyadic regulation

## Abstract

**Background:**

Face-to-face visual contact is a key component of the early parent-child interaction, therefore a visual impairment condition of the parent or the child represents a risk factor for dyadic patterns' development.

**Aims:**

The study presents a critical single case of a blind father and a 18-month-old visually impaired child. The study aims to explore changes in the relational functioning of this dyad during an early family-centered intervention.

**Methods and procedures:**

Ten parent-child sessions were videotaped and micro-analytically coded. Data were analyzed through a State Space Grid crossing child's social cues and types of father verbalizations.

**Outcomes and results:**

Findings showed a stable increase in the amount of child social cues over time. Moreover, the dyad exhibited progressive changes in dyadic regulation, stability, and organization. The return time to the “*active interaction*” region of interest decreased progressively. A reduction was observed also for the time spent by the dyad in the region “*no vocal contact*.”

**Conclusions and implications:**

This critical single case highlighted the benefits of parental engagement in early interventions for the dyadic regulation in parent-child interaction.

## Introduction

The early experience of reciprocal interactive patterns with the caregivers is key for children's cognitive, social, and affective development (DiCorcia and Tronick, 2011; Henning and Striano, 2011; Müller et al., 2015). The parent-child dyad has been described as an open, non-linear, and multi-final dynamic system (Smith and Thelen, 2003). This dyadic system grows over time in complexity and organization, develops stable functional patterns (i.e., attractor states), and still maintains enough flexibility to allow timely and adaptive responses to environmental stimulations or perturbations (Lewis et al., 1999; Grumi et al., 2022). Dyads who are capable of developing stable and recurrent behavioral patterns and still are relatively flexible to adapt to environmental changes allow the child to create reliable expectations about his/her relational world (DiCorcia and Tronick, 2011).

Measures of dyadic flexibility and organization are available from videotaped parent-child interactions by using the States Space Grid (SSG; Hollenstein et al., 2004). This is a quali-quantitative tool to study parent-child functioning and it provides a grid representation of the dyadic space defined by the categorical or ordinal coding scheme of each interactive partners' behavior. By crossing the individual behavioral categories of the parent and the child, a grid space is obtained and it allows to plot the trajectories of dyadic states in time (Hollenstein et al., 2004). A growing body of literature provided evidence that the SSG may be specifically advantageous to the study of parent-child interactions as highlighted in a recent systematic review (Grumi et al., 2022).

Face-to-face visual contact is a key component of the early parent-child interaction, at least in typically developing children. Indeed, most of the early human dyadic interactions typically rely on visual feedback, visual joint attention, and gaze following (Farroni et al., 2002; Jongerius et al., 2020). One might wonder whether and how children or parents with visual impairment develop dyadic patterns of organization and flexibility. Children with low-vision or complete blindness conditions may lack—at least partially—the capacity to timely respond to vision-dependent requests or behaviors (Adamson et al., 1977). Eye contact, visual imitation, recognition of emotional expressions, and cognitive-spatial orientation may be impaired, and this may dramatically limit opportunities to engage in social relationships (Dale et al., 2014; Damen et al., 2015). A recent systematic review of this literature (Grumi et al., 2021) highlighted that responsivity to interactive stimuli may be the most impaired domain of early dyadic interactions in these children. In addition, visually impaired children might exhibit less frequent smiles or social vocalizations and they may not react contingently to parents' requests or verbalizations (Rogers and Puchalski, 1984; Rowland, 1984; Nagayoshi et al., 2017). Finally, a reduction in the endogenous production of intentional social cues in the interaction has been observed in previous studies (Tröster and Brambring, 1992; Conti-Ramsden and Pérez-Pereira, 1999).

Parent's visual impairment might also affect the quality of the early caregiver-child interaction. Nonetheless, the effects of parental visual loss on parent-child interaction has received less attention in previous literature. Decades ago, Lauren Adamson has shown how parents' sensory impairment may expose the child to repetitive violations of social expectations in daily face-to-face interactions (Adamson et al., 1977). For example, these caregivers cannot detect child's visual cues and they may fail to contingently respond to child's non-verbal signals. These difficulties may lead to longer periods of “interactive silence” characterized by an absent vocal contact, which—in absence of visual contact—may completely impair the possibility of the interactive partners to detect and be aware of each other behaviors and mental states. However, some studies highlighted that the parents who are blind may use compensative strategies: for example, blind parents have been found to rely on other senses (e.g., instrumental touch) to monitor the child explorative behaviors during play sessions (Collis and Bryant, 1981). Notably, the presence of parents' visual impairment did not necessarily imply deficits in child's development. Indeed, previous studies showed that sighted infants of blind parents may exhibit high flexibility in early communicative behaviors (Ganea et al., 2018) and typical development of gaze processing and socio-communicative skills has been reported in these children (Senju et al., 2013).

In this scenario, early interventions focused on parent-child interaction are priority to support both sensitive parenting and child's development in the presence of developmental visual impairment (Elsman et al., 2019). In this paper, we report on a critical single case characterized by a double risk condition for the parent-child interaction quality: the visual impairment of the child and the blindness of the father. We present this case as the dyad proceeds in its regulatory attunement as a system across a ten-session early intervention conducted at the IRCCS Mondino Foundation Developmental Neuro-ophthalmology Unit (Morelli et al., 2020). The micro-analytic coding of the dyadic exchange was carried on to highlight the progressive change in the relational functioning of a dyad who face a double-risk condition.

## A critical single case

### Clinical presentation

Adam^1^ was referred to the Developmental Neuro-ophthalmology Unit when he was 15 months by an ophthalmologist specialist on suspicion of Leber's congenital amaurosis—a very early onset type of retinal dystrophy, genetically determined and characterized by a severe hypovision or blindness from the first months of life. It was reported that Adam's mother also suffered from the same condition. At the first outpatient visit, Adam was accompanied by his father, blind due to retinopathy of prematurity (ROP). It was reported that from the age of 5 months the family had begun to notice Adam's tendency to “keep the eyes down” and his difficulty in visual engagement: the eye examination carried out at the age of 6 months found a pale optic papilla, attenuated retinal vessels and absence of retinal pigment, posing the clinical doubt of retinal dystrophy. The doubt of retinal pathology was later confirmed by electrophysiological examinations (visual evoked potentials, electroretinogram), which documented a very advanced photoreceptor impairment and conduction delay.

### Clinical assessment and intervention

**Figure 1:**
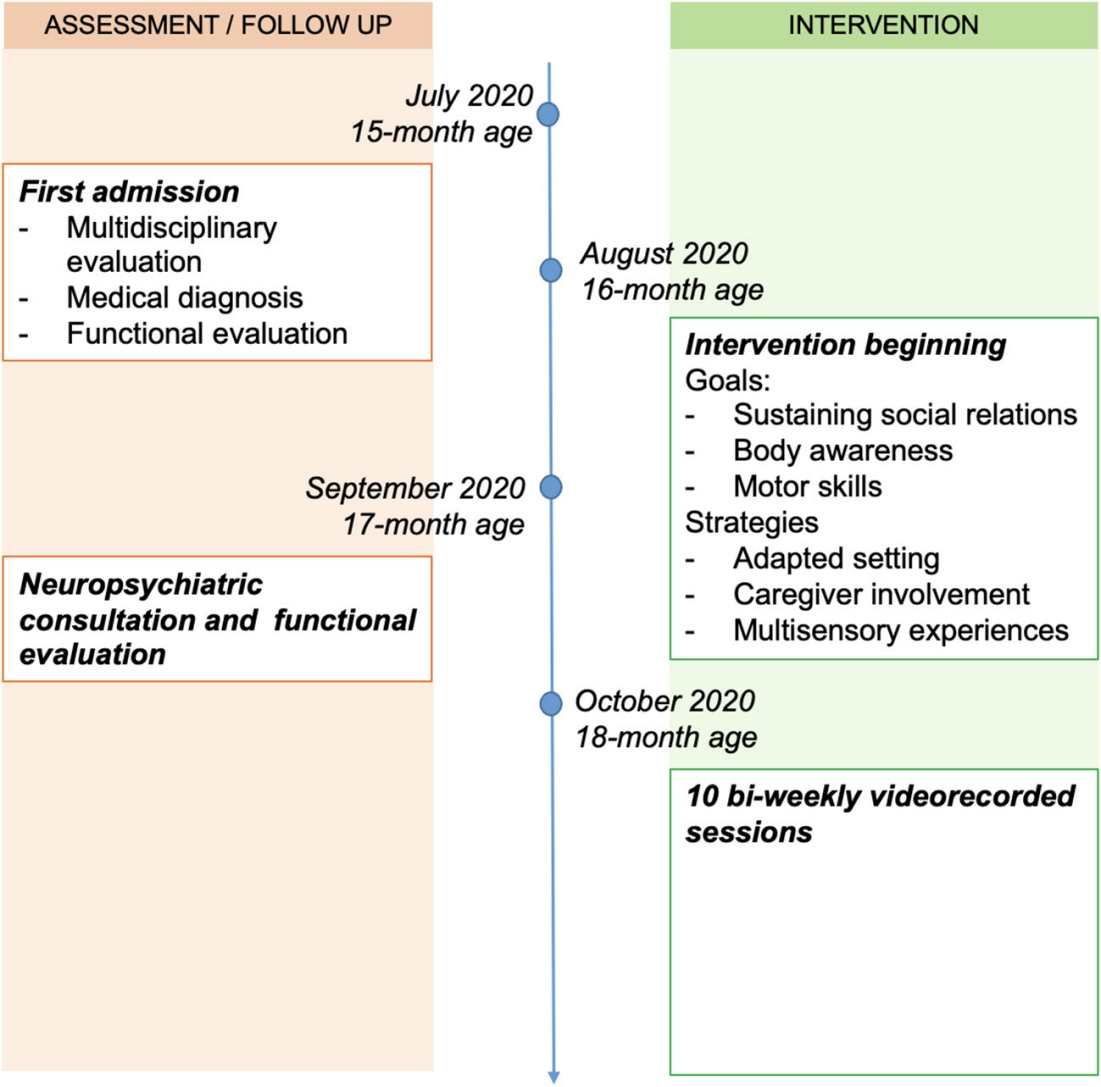
Timeline of the case clinical assessment and intervention.

Details on the assessment procedures and intervention are reported in Supplementary File 1. In the present manuscript, we report on the first 10 bi-weekly sessions of an outpatient intervention. Each session lasted 1 h and 30 min and it occurred in a dedicated playroom. During each session, the father and the child were always present in the playroom together with a psychomotor therapist. The research assistant who videotaped the father-child interaction was in the room only for the time needed to acquire the video. The timeline of the case clinical assessment and intervention is reported in Figure 1.

### Ethics

The father provided informed consent to participate in the data collection and agreed for these data to be published in the present paper. The information on the child and his father have been reduced to a minimum amount to balance the need to give readers a context for these observations and to avoid any identifying detail. The name of the child has been changed to Adam.

## The use of the SSG in a clinical setting

### Data collection and coding

For each intervention session, the father and the child were asked to interact for 5 min with no interruptions from the therapist. They were given a set of toys chosen by the therapist based on the clinical profile of the child and the intervention aims of the session (see Additional materials). The videotaped interaction was than coded off-line by a trained research assistant. The 2 s micro-analytical coding was performed using Elan 5.9 for Windows 10 (Lausberg and Sloetjes, 2009). The coding scheme is reported in Table 1.

**Table 1:** Coding scheme.

**Interactive partner**	**Description**	**2 s coding levels**
**Child**
Social cues	Any vocal (e.g., calling the father, commenting, or making a request) or gestural (e.g., passing an object to the father, reaching out, clinging, reaching physical proximity) social communication of the child directed to the father	1 = Yes (presence of at least one social cue) 0 = No (absence of social cues)
**Parent**
Verbalizations	Any verbalization of the father directed to the child	1 = No (the father was silent)
		2 = Neutral (any verbalization which had no child-directed social meaning, like coughing or unspecified verbalizations)
		3 = Cognitive (verbalizations that had specific cognitive goals such as requests directed to the child, attention getting verbalizations, and explanations about objects or sequences of actions)
		4 = Affective (verbalizations that had positive, affective meaning such as mirroring child vocal productions, positively commenting on the child mental or behavioral state, using affective nicknames, and saying something positive approving child's behavior)

### Dyadic measures

**Figure 2:**
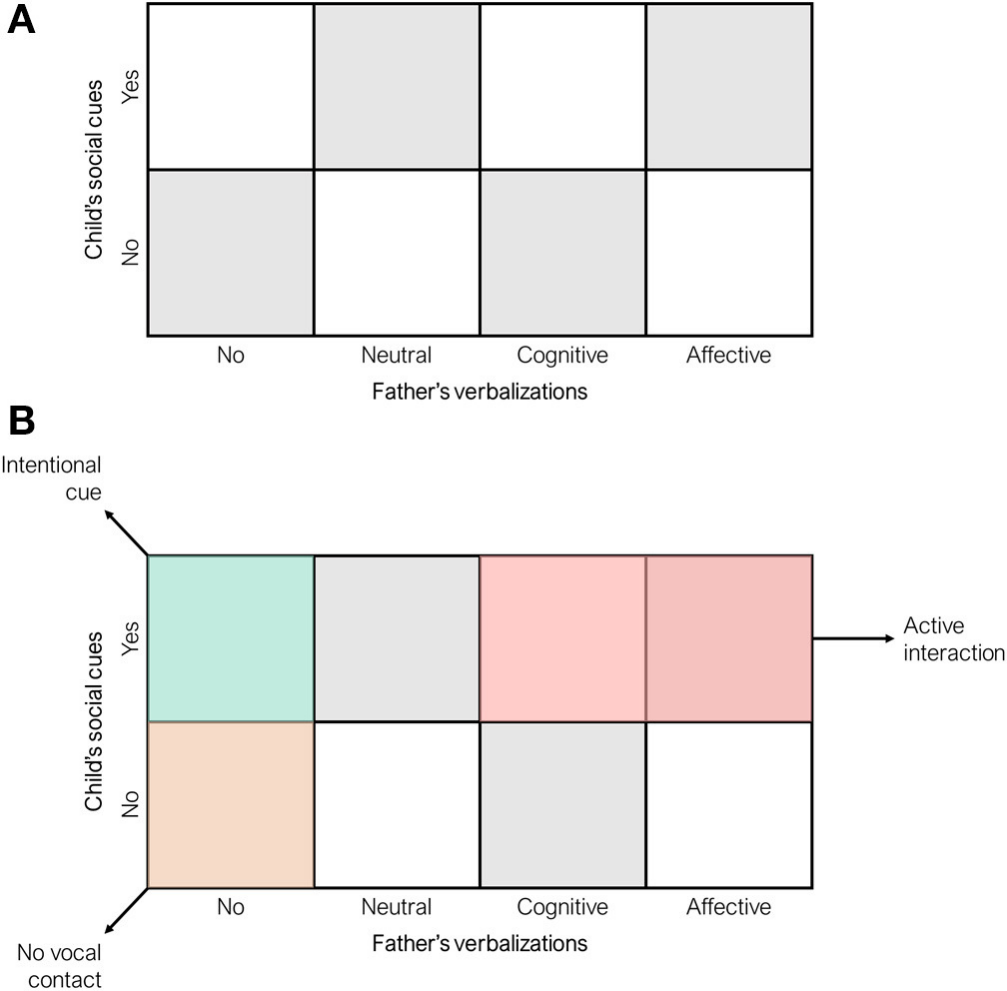
State Space Grid (SSG) cells **(A)** and regions of interest **(B)** used in the present study.

According to the coding scheme reported in Table 1, we were able to obtain for each of the ten videotaped interactive sessions a 2 × 4 SSG representation defined by child's social cues on the y-axis (2 levels: yes; no) and the father verbalizations on the x-axis (4 levels: silent; neutral; cognitive; affective). A blank example of this SSG is reported in Figure 2A. In the SSG graphical representation, each dot is a dyadic state event, lasting at-least 2 s based on the micro-analytic time unit adopted in this study. Moreover, a visit is a prolonged stay in a specific dyadic state which can be made by two or more dyadic state events.

#### Content-free and content-specific measures

For the purposes of the present study, two content-free SSG measures were obtained for each session: range and duration per cell (flexibility) (see Table 2). Target cells were also identified to obtain content-specific measures. The target cells are represented in Figure 2B and described in Table 3.

**Table 2:** Content-free dyadic measures.

**Measure**	**Description**	**Range**	**Description**
Range	Count of the State Space Grid cells visited during the entire interaction by the dyad	1–8*	Low range values are meant to reflect the presence of a rigid dyadic functioning characterized by a limited exploration of the potential dyadic states, whereas high range values suggest the presence of more fluid and dynamic regulation of dyadic exchanges in the interaction
Duration per cell	Sum of the total seconds spent by the dyad in a specific cell	37.5–300 s**	Low values of this variable reflect the tendency of the dyad to move freely among the possible dyadic states (high flexibility), whereas high values are an index of the dyadic tendency to be still in a limited number of dyadic states (low flexibility or high stickiness)

*This range simply reflects the min and max number of dyadic states in a 2 × 4 State Space Grid. **This range is obtained considering that the total available time per session was 300 s and that the max number of dyadic states was 8 (300/8 = 37.5).

**Table 3:** Content-specific dyadic regions of interest and measures.

**Label**	**Description**	**Number of cells**	**Measures**
Active interaction	Dyadic state resulting by the co-occurrence of child social cues and father's cognitive or affective verbalization	2	Return time: number of 2 s events needed for the dyad to return to this SSG area after moving to other dyadic states
Intentional cue	Dyadic state resulting by the co-occurrence of child social cues and no father's verbalization (silent)	1	Duration per visit: mean number of consecutive 2 s events
No vocal contact	Dyadic state resulting by the co-occurrence of no child social cues and no father's verbalization (silent)	1	Total time: maximum time spent by the dyad during each session

### Individual and dyadic functioning across the intervention

**Figure 3:**
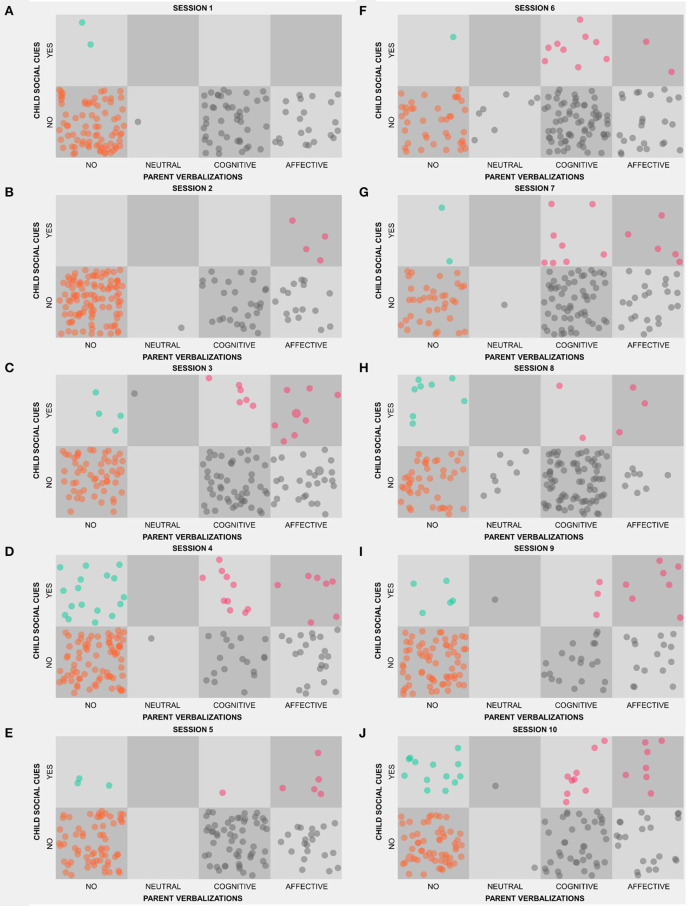
State Space Grid (SSG) representations of the father-child dyadic interaction from session 1 **(A)** to session 10 **(J)**.

The SSG representation from session 1 to session 10 is reported in Figures 3A–J. The individual ratings for Adam and his father are reported in the additional materials.

**Figure 4:**
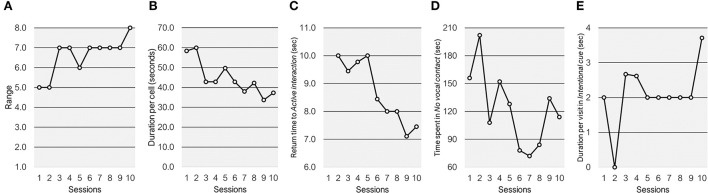
Whole grid and region of interest measures across the ten sessions. **(A)** Range; **(B)** duration per cell; **(C)** return time to the active interaction region; **(D)** time spent in the no vocal contact cell; **(E)** duration per visit in the intentional cue cell.

From a dyadic perspective, the range of dyadic states explored by the interactive couple gradually increased from five during session 1 to the maximum number of eight during session 10 (see Figure 4A). The whole grid duration per cell decreased prominently from session 1 (58.4 s) and session 2 (60 s) to mid-interventions sessions 5 and 6 (respectively, 49.7 and 42.9 s) and to the final sessions 9 and 10 (respectively, 33.7 and 37.3 s) (Figure 4B).

The return time to the *active interaction* region of interest decreased was not computed during sessions 1, as there was no occurrence of this dyadic state. It was relatively stable from session 2 to session 5, ranging from 9.5 to 10 s. From mid-intervention to session 10 the return time to *active interaction* decreased progressively below 8 s during sessions 9 and 10 (Figure 4C). A reduction was observed also for the time spent by the dyad in *no vocal contact*. Whereas, this dyadic state was relatively highly frequent during the first two sessions (i.e., time spent higher than 2.5 min during the 5 min interaction), it gradually reduced below 1.5 min sessions 6-yo-8, with a minimum of 72 s during session 7 (Figure 4D). Although time spent in *no vocal contact* increased during sessions 9 and 10, it remained below the 50% of the total interaction length. The duration per visit for the *intentional cue* dyadic state was relatively stable across the sessions and the child only rarely show a mean duration higher than 2 s (i.e., one coding event) from sessions 1–9 (Figure 4E). Only during sessions 10, Adam showed greater persistency of intentional communications toward the father, with an average mean duration of visit in the *intentional cue* dyadic state approaching the value of 4 (i.e., two consecutive coding events).

## Discussions and implications

The present article reports on a critical single case of a family-centered rehabilitation intervention involving an 18-month-old child with visual impairment and his blind father. The intervention lasted for 10 bi-weekly sessions and 5-min videotapes of father-child interaction were obtained at the beginning of each session for off-line coding and SSG analyses. The single case suggested that across a relatively short time (i.e., ~5 months) stable increases in the amount of child social cues were achieved. Moreover, the parent-child dyad also exhibited progressive changes in dyadic regulation, stability, and organization.

First, two content-free SSG measures (i.e., range and duration per cell) suggested that the dyadic system composed by Adam and his father progressively increased the amount of dyadic states experienced during the play interaction. These findings highlight the possibility that a relatively brief family-centered approach to the rehabilitation of children with visual impairment may also result in relevant adaptations of the parent-child system. Previous research has highlighted that parents and children with visual impairment may experience specific barriers and challenges in achieving reciprocally satisfaction and attunement in daily interactions (Grumi et al., 2021). Parents' understanding of the child social cues may be partially impeded and the child may present deficiencies in the ability to engage and contingently respond to parents' requests and proposals (Sakkalou et al., 2021). At the same time, it is well-known that parenting represents the large majority of the caregiving environment and even when the child presents visual impairment parents can succeed in providing appropriate support to their child to achieve exploration, cognitive, and emotional milestones (Dale and Salt, 2007; Dale et al., 2019). Extending previous evidence, this clinical case further underlines that it is not only child behavior that benefits from an early intervention; rather, it is the entire parent-child system that re-organize to embed child rehabilitation achievements. This single case is far more intriguing as both the child and the father presented moderate to severe visual impairment.

When looking at specific content-related dyadic states, quantitative increases were observed for active communication and intentional cues. The first dyadic state was characterized by a conjoint production of socially relevant communications by both the interactive partners. In the 2 s time unit considered in this study, the child is making social cues directed to the father, while the father is making a cognitive or affective verbalization. For example, the child may bring an object to the father who says its name or the father may call the child and he responds contingently in a time frame of 2 s. This kind of active engagement between the father and the child visibly increases already during the first sessions and remains consistently high across the following nine sessions. Nonetheless, the increase in the intentional cues also highlights that the amount of time spent by the dyad in a state characterized by child active communications and father's attendance to these signals is positively affected by the intervention. In other words, it is possible to speculate that as this intervention promotes better social skills and communication capacities in the child with visual impairment in the presence of the father, the father may indirectly benefit from an increasing awareness of his child as a social agent. Unfortunately, in the present study we did not collect any information on the fathers' mental representations of himself as a parent and of his child. A specific investigation of how an early intervention may change the mental representation that parents' have of their children is warranted to be a promising goal for future research in this field.

Lastly, and consistently with the findings reviewed above, the time spent by the dyad in no vocal contact decreased from session 1 to session 10. This means that the dyad was gradually much more actively engaged in reciprocal and contingent social, cognitive, and affective exchanges as the intervention progressed. It should be noted here that the present critical single case presented a double-risk condition as both the child and the father had a visual impairment. As such, for this dyad, the absence of vocal contact was not counterbalanced by visual engagement as it may occur in dyads of parents and children with no visual deficits. For Adam and his father, being silent means being absent for most of the times. From this perspective, while the child was becoming more and more able to produce social cues based on his intentional need to engage the adult, the father was also able to recognize Adam's social communications and to respond contingently with cognitive or affective comments. As reported in Table 1, it should be also highlighted that while a quantitative increase in social cues was evident for the child, the father did not show any specific increase in any of the verbalization types across the intervention. In other words, this single case is consistent with the theoretical claim of the dynamic system theory related to the contribution of a behavioral change in one individual affecting the dyadic regulation of the entire system (Provenzi et al., 2018). Here, the change in Adam's behavior (e.g., increase in social cues) reflects in dyadic re-arrangement during the intervention even in the absence of specific changes in the caregiving behavior of the father.

Of course, mechanisms of change here can only be hypothesized. In absence of fathers' visual access to what the therapist was doing with the child—e.g., the kind of proposals, the speed and direction of gestures, the type of objects involved, etc.—the father may have been able to obtain information by directly discussing with the therapist or using other senses (e.g., physical contact) to interpret and give new meanings to his child movements and actions. Additionally, it is also possible that implicit knowledge and learning may be in place here and this may link the behavioral and socio-cognitive achievements of Adam with contingent adaptations in the caregiving mind-set of his father. Anecdotical evidence from the sessions seem to support this hypothesis. Fathers' comments such as “I didn't know you would like this” or “I did not expected you to be able in doing this” may indicate that the father is changing the mental representation he has of his child abilities while the intervention proceeds.

This study has limitations. First of all, this is a critical and unique single case. One can speculate how such an intervention might work when the clinical and sensory profiles of the parent are less impaired (Dale et al., 2019). Additionally, the micro-analytical coding used for paternal verbalizations was focused on the semantic language dimension, while prosodic aspects were not evaluated (Nelson et al., 1989; Gupta et al., 2016). This study did not include other parental gestural behaviors like touch, however investigating the use of other sensory channels (i.e., tactile) and multimodal stimulations when interacting with visually impaired children is crucial and represents a future direction of studies in this field.

## Conclusion

A family-centered approach to the rehabilitation program of children with visual impairment is highly recommended. The present critical single case highlights that the benefits of engaging the parents in the early intervention may not only limit to the child growth and developmental achievements; rather, they may extend to improve the dyadic regulation of parent-child interaction, which is a major part of the caregiving environment experienced by the child in daily life. By facilitating the emergence of more flexible, yet organized and functional systemic set-up of the parent-child dyad, a family-centered approach to child rehabilitation in developmental vision impairment thus appears to be a valid and powerful strategy. While future studies with controlled trial designs may give quantitative and parametric estimates of the efficacy of this intervention, this paper clearly highlights that the active engagement of parents in the early rehabilitative intervention of young child with sensory impairment should not be considered accessory and family-centered care should be prioritized.

## Author's note

This paper presented a critical single case characterized by a double risk condition: the visual impairment of the child and the blindness of the father. Literature about how blindness and visual impairments may impact the quality of parent-child interactions is limited and sparse. No studies in our knowledge investigated dyadic interactions in presence of a visual impairment of both the parent and the child. Moreover, fathers' engagement in early parent-child interventions has received far less attention in scientific literature. From a methodological point of view, this study used an innovative quali-quantitative approach to analyze the parent-child interaction: States Space Grids (SSG). The SSG tool provides graphical representations and quantitative assessments of different measures of dyadic flexibility and organization, therefore it is specifically advantageous to the study of parent-child interactions. Findings about dyadic regulation changes in this parent-child dyad during an early family-centered intervention have relevant theoretical and clinical implications. On one hand, these findings deepen the knowledge about the relevance of visual channel for the development of child's socio-cognitive skills. On the other hand, they highlight the parental engagement in early interventions.

## Data availability statement

The raw data supporting the conclusions of this article will be made available upon reasonable request to the corresponding author.

## Ethics statement

The studies involving human participants were reviewed and approved by Ethics Committee Pavia. Written informed consent to participate in this study was provided by the participants' legal guardian/next of kin.

## Author contributions

LP and SG conceptualized the study. AL, DP, GA, FM, EM, and SS contributed to data collection. GP and LV contributed to the data analysis. LP drafted the first version of the manuscript. All the authors agreed upon the submission of the final version of the manuscript.

## Funding

This study was supported by funds from the Italian Ministry of Health: Cinque per Mille, 2017 (LP) and Ricerca Finalizzata Starting Grant SG-2019–12369732 (SG).

## Conflict of interest

The authors declare that the research was conducted in the absence of any commercial or financial relationships that could be construed as a potential conflict of interest.

## Publisher's note

All claims expressed in this article are solely those of the authors and do not necessarily represent those of their affiliated organizations, or those of the publisher, the editors and the reviewers. Any product that may be evaluated in this article, or claim that may be made by its manufacturer, is not guaranteed or endorsed by the publisher.

## Footnotes

^1^Adam is a fictional name to guarantee the patient anonymity.
