# Supplementary material for: Case report: Dancing in the dark: A critical single case study engaging a blind father in the rehabilitation journey of his visually impaired child
Source: Front Psychol. 2022 Oct 13;13:942321. doi: 10.3389/fpsyg.2022.942321 (PMC9606581; doi:10.3389/fpsyg.2022.942321)
Supplement: Supplementary file 1 [file Data_Sheet_1.docx]

**Manuscript title |** Dancing in the dark: A critical single case study engaging a blind father in the rehabilitation journey of his visually impaired child

**Authors |** Provenzi et al

**Doi |** 10.3389/fpsyg.2022.942321

**Supplementary File S1**

1. *Clinical assessment*

Gathering information about visual function in daily living activities was challenging given the parents' significant visual impairment. Only a prevalent difficulty in distance vision and a slightly more effective perception of objects from near distance were described. No significant motor impairments were reported. The multidisciplinary evaluation of visual function, carried out by a child neuropsychiatrist and a psychomotor therapist, revealed a picture of severe visual impairment (binocular grating acuity was tested with Teller Acuity Card and only Low Vision Card was detected; Teller et al., 1986) with only occasional visual engagement, a little more effective in low light environments or for high-contrasted targets. On neurological physical examination, neuromotor findings were consistent with age and there were no associated neurological symptoms. During this first visit, Adam showed himself to be a sufficiently attentive and curious child towards the environment and the people around him, reactive to his father's voice, and quite tolerant of physical contact. His mimicry was poor, but over the course of the visit Adam was able to smile at the examiners on some occasions. As a result of this first assessment, it was agreed with the family to start a rehabilitation program at our center, specifically focused on neurovisual aspects, and aimed at promoting global development. The intervention started at the age of 16 months. By the time this paper is written, the diagnosis of Leber congenital amaurosis was genetically confirmed.

1. *The philosophy of the intervention*

The Developmental Neuro-ophthalmology Unit of the IRCCS Mondino Foundation, Pavia (Italy), adopts family-centered and multi-disciplinary approach to the rehabilitation of visually impaired children. This approach maximizes the collaboration among professionals and parents (Morelli et al., 2020). At first admission, a comprehensive clinical assessment is performed to identify the patient’s clinical profile. Then, the rehabilitative aims and strategies are set in a tailored way based on the most damaged patient’s visual subsystems (i.e., primary visual pathway, oculomotor system, and associative visual areas) (Morelli et al., 2020). For the specific case of very young infants and children, the rehabilitative activities largely rely on collaborative play sessions where the parents and the therapists alternate in the interaction with the child to identify the best rehabilitative options to support social, affective, and cognitive development of children with visual impairment conditions. During these sessions, all the methodological and clinical choices of the therapist are tailored on the child needs and resources and they are discussed with the parents to maximize the possibility to expand what is learned during the rehabilitation session to the home environment.

1. *Intervention details*

The intervention took place at the Developmental Neuro-ophthalmology Unit of the IRCCS Mondino Foundation, Pavia (Italy) and it is described in detail in a previous publication from this group (Morelli, Aprile, Cappagli, Luparia, Decortes, Gori et al., 2020). This clinical deals with different types of visual disorders and provides both diagnostic and re-habilitation services. The intervention is coordinated by a child neuropsychiatrist and engages a multi-disciplinary professional team (i.e., psychomotor therapists, psychologists, rehabilitation technicians, orthoptists, and ophthalmologists). It is articulated in three major steps: first, a comprehensive clinical assessment is performed and provides a complete clinical profile of the young patient (e.g., etiology of the visual impairment, functional vision, psychomotor development, and neuropsychiatric aspects); second, depending on the clinical profile obtained, the most damaged visual subsystem is identified; third, specific re-habilitation strategies are promoted consistent with the clinical profile and the most damaged visual subsystem. A core dimension of the intervention is the active engagement of parents throughout all the steps. The parent is always present in the observation and intervention room and is considered an active partner of the re-habilitation team. Support to parenting and to the parent-child relationship is promoted by providing parents with an educational modeling, favoring parents’ loud-voice comments on the child’s behavior, by promoting a collaborative discussion with the therapist, and by sharing the ongoing knowledge on the child’s abilities and difficulties while dealing with the social and the physical word during the sessions. Specific sensory games are discussed with the parent, so that they can be replicated at home between consecutive sessions. Moreover, psychological support is offered to allow parents’ accepting their own child’s disability and to promote parental awareness of the child’s needs, communicative signals, and everyday challenges.
